# Magnetic resonance imaging based kidney volume assessment for risk stratification in pediatric autosomal dominant polycystic kidney disease

**DOI:** 10.3389/fped.2024.1357365

**Published:** 2024-02-23

**Authors:** Kubra Yilmaz, Seha Saygili, Nur Canpolat, Ozlem Akgun-Dogan, Zeynep Nagehan Yuruk Yildirim, Rumeysa Yasemin Cicek-Oksuz, Huseyin Adil Oner, Bagdagul Aksu, Nazli Gulsum Akyel, Ozge Oguzhan-Hamis, Hasan Dursun, Sevgi Yavuz, Neslihan Cicek, Nurver Akinci, Esra Karabag Yilmaz, Ayse Agbas, Ahmet Nevzat Nayir, Dildar Konukoglu, Sebuh Kurugoglu, Lale Sever, Salim Caliskan

**Affiliations:** ^1^ Department of Pediatrics, Cerrahpasa Faculty of Medicine, Istanbul University-Cerrahpasa, Istanbul, Türkiye; ^2^ Department of Pediatric Nephrology, Cerrahpasa Faculty of Medicine, Istanbul University-Cerrahpasa, Istanbul, Türkiye; ^3^ Division of Pediatric Genetics, Department of Pediatrics, Acıbadem University School of Medicine, Istanbul, Türkiye; ^4^ Department of Pediatric Nephrology, Istanbul Faculty of Medicine, Istanbul University, Istanbul, Türkiye; ^5^ Department of Pediatric Nephrology, Istanbul Bakirkoy Sadi Konuk Hospital, Istanbul, Türkiye; ^6^ Department of Pediatric Basic Sciences, Istanbul University, Institute of Child Health, Istanbul, Türkiye; ^7^ Department of Pediatric Radiology, Cerrahpasa Faculty of Medicine, Istanbul University-Cerrahpasa, Istanbul, Türkiye; ^8^ Department of Pediatric Nephrology, Istanbul Prof. Dr. Cemil Tascioglu City Hospital, Istanbul, Türkiye; ^9^ Department of Pediatric Nephrology, University of Health Sciences, Istanbul Basaksehir Cam and Sakura City Hospital, Istanbul, Türkiye; ^10^ Department of Pediatric Nephrology, Marmara University School of Medicine, Istanbul, Türkiye; ^11^ Department of Pediatric Nephrology, Bezmialem Vakif University Hospital, Istanbul, Türkiye; ^12^ Department of Biochemistry, Cerrahpasa Faculty of Medicine, Istanbul University-Cerrahpasa, Istanbul, Türkiye

**Keywords:** ABPM, ADPKD, children, hypertension, kidney volume, magnetic resonance imaging, (MRI), pediatric

## Abstract

**Introduction:**

In the pediatric context, most children with autosomal dominant polycystic kidney disease (ADPKD) maintain a normal glomerular filtration rate (GFR) despite underlying structural kidney damage, highlighting the critical need for early intervention and predictive markers. Due to the inverse relationship between kidney volume and kidney function, risk assessments have been presented on the basis of kidney volume. The aim of this study was to use magnetic resonance imaging (MRI)-based kidney volume assessment for risk stratification in pediatric ADPKD and to investigate clinical and genetic differences among risk groups.

**Methods:**

This multicenter, cross-sectional, and case-control study included 75 genetically confirmed pediatric ADPKD patients (5–18 years) and 27 controls. Kidney function was assessed by eGFR calculated from serum creatinine and cystatin C using the CKiD-U25 equation. Blood pressure was assessed by both office and 24-hour ambulatory measurements. Kidney volume was calculated from MRI using the stereological method. Total kidney volume was adjusted for the height (htTKV). Patients were stratified from A to E classes according to the Leuven Imaging Classification (LIC) using MRI-derived htTKV.

**Results:**

Median (Q1-Q3) age of the patients was 6.0 (2.0–10.0) years, 56% were male. There were no differences in sex, age, height-SDS, or GFR between the patient and control groups. Of the patients, 89% had PKD1 and 11% had PKD2 mutations. Non-missense mutations were 73% in PKD1 and 75% in PKD2. Twenty patients (27%) had hypertension based on ABPM. Median htTKV of the patients was significantly higher than controls (141 vs. 117 ml/m, *p* = 0.0003). LIC stratification revealed Classes A (38.7%), B (28%), C (24%), and D + E (9.3%). All children in class D + E and 94% in class C had PKD1 variants. Class D + E patients had significantly higher blood pressure values and hypertension compared to other classes (*p* > 0.05 for all).

**Discussion:**

This study distinguishes itself by using MRI-based measurements of kidney volume to stratify pediatric ADPKD patients into specific risk groups. It is important to note that PKD1 mutation and elevated blood pressure were higher in the high-risk groups stratified by age and kidney volume. Our results need to be confirmed in further studies.

## Introduction

Autosomal dominant polycystic kidney disease (ADPKD) stands as one of the most prevalent hereditary kidney disorders, marked by the progressive development of kidney cysts, hypertension (HT), and eventual progression to end-stage kidney disease (1, 2). Among individuals with ADPKD, cardiovascular complications emerge as the leading cause of mortality, with hypertension serving as a key risk factor for cardiovascular disease (3, 4).

In the pediatric presentation of ADPKD, a distinctive aspect emerges in which hyperfiltration maintains the glomerular filtration rate (GFR) within normal limits despite underlying structural kidney damage (5). It is noteworthy that detecting a decline in GFR during adulthood signifies irreversible damage, underscoring the critical necessity for early intervention. Therefore, there is undoubtedly need for biomarkers with enhanced predictive power to facilitate timely initiation of treatment and prevent irreversible damage (6).

The Consortium for Radiologic Imaging Studies of Polycystic Kidney Disease (CRISP) cohort study has revealed a noteworthy inverse correlation between kidney volume measured by magnetic resonance imaging (MRI) and kidney function in individuals with ADPKD aged 15 years and older (7). Adult-focused studies highlighted the importance of height-corrected kidney volume (htTKV) as a excellent predictor of renal dysfunction (8–11).

Existing risk stratification methods, like the Mayo Imaging Classification (MIC), have provided a framework for adult patients that incorporates age and MRI-measured htTKV. However, these classifications are only applicable to individuals aged 15 years and older. Moreover, the prediction models of htTKV increase shows great discrepancies before the adult age, underlines the importance of exploring the pediatric htKTV change (12). More recently, the utility of these classifications has been adapted to the pediatric population as the Leuven Imaging Classification (LIC), using three-dimensional ultrasound (3DUS) for kidney volume measurement (13).

Ultrasound volumetry tends to underestimate kidney volume in ADPKD compared to MRI; however, three-dimensional ultrasound (3DUS) with manual contouring has demonstrated superior accuracy over traditional two-dimensional ultrasound. Therefore, 3DUS applied with a correction factor stands as a promising alternative to MRI with a lower cost and burden in children (14). However, MRI remains the gold standard for TKV quantification, but its use is often perceived as time-consuming and may require sedation in children.

The aim of the present study was to use MRI-based volume in children and the novel LIC in pediatric ADPKD patients to stratify them into specific risk groups. Our primary focus is to investigate clinical, genetic, and ambulatory blood pressure (ABPM) differences among children with ADPKD stratified according to the LIC and identify children at increased risk of disease progression.

## Materials and methods

### Study design and population

This multicenter, cross-sectional, and case-control study included 89 children and adolescents (39 girls, 50 boys) with a diagnosis of ADPKD (*patient group*), and 27 age- and sex-matched healthy children (13 girls, 14 boys) (*control group*). The exclusion criteria were as follows: patients (i) having no genetical diagnosis, (ii) younger than 5 years of age or older than 18 years of age at the study time, (iii) any other disease affecting kidney function or anatomy, (iv) not suitable for MRI (as having dental braces or pacemaker), and (v) patients or their parents who did not agree to participate in the study. Finally, 75 children with ADPKD were eligible for inclusion in the study. The cross-sectional evaluation of the study was carried out between May 2019—November 2020, after the approval of the study by the Ethics Committee of the University of Istanbul University-Cerrahpaşa (58213/ 12.04.2019). All examinations of the patients were performed in accordance with good medical and laboratory practices and the recommendations of the Declaration of Helsinki on Biomedical Research Involving Human Subjects. The patient and control groups and their families were informed about the study procedure. Informed consent was obtained from each eligible patient or their parents/caregivers.

Medical history was collected from the patients' files. Anthropometric and blood pressure (BP) measurements and MRI scans were performed at the same time. Anthropometric data [weight, height, and body mass index (BMI)] were adjusted according to the Turkish normative values and expressed as their SDS values (15). Obesity defined as BMI >  + 2 SD for height–age (16).

### Genetic analysis

After obtaining written informed consent, peripheral blood samples were collected from patients. Following the standard protocols of the QIAAmp DNA Mini (*Qiagen*) kit, DNA was automatically isolated in EDTA-anticoagulated blood samples. Targeted NGS analysis was performed using Nephropathies Panel by Sophia Genetics kit, a custom panel using a capture-based method. Virtual renal cysts gene panel was created consisted of 10 genes; PKD1, PKD2, PKHD1, HNF1B, CEP290, EYA1SIX1, OCRL, UMOD, TTC21B were analyzed. Illumina MiSeq (v1.9) was used as the sequencing platform. Sequence analysis covers coding regions of each gene, including all coding exons, ±10 base pairs of adjacent intronic sequences, and each nucleotide is read at a depth of at least 50X. Any variants that fall outside these regions and exonic variants with a minor allele frequency of less than 10% were considered as false positives and not analyzed. Copy number variations (CNV) were also examined. The DNA sequences were aligned to the NCBI Build37 (hg18) version of the human genome. The Sophia-DDM-V5.2 bioinformatics analysis program performed variant calling and data analysis. The interpretation of the variants was performed according to the 2015 American College of Medical Genetics (ACMG) standards and guidelines (17). Since there are not enough genome and exome databases for the Turkish population, Iranome, GnomAD data were used as the control population. The variants effects on protein function were investigated using in-silico prediction programs such as SIFT, MutationAssessor, MutationTaster, and MVP. Human Gene Mutation Database (HGMD) and ClinVar database were used to investigate mutations previously reported.

Variants were classified into five categories: benign (B), likely benign (LB), variant of unknown significance (VUS), pathogenic (P), likely pathogenic (LP). For P, LP, and VUS variants, the variant type, zygosity, variant location, HGMD accession number, ACMG variant classification, and evidence used for variant classification are shown in Supplementary Table S1. LB or B variants are not shown. Segregation analysis was performed by Sanger sequencing. Primer sequences and reaction conditions are not shown (data available on request).

### Kidney functions assessment

Blood samples for the measurements of creatinine and cystatin C were obtained in the morning after an overnight fast. Serum cystatin C concentrations were measured by the immunonephelometric method (*Siemens, Atellica® NEPH 630 System/BN II System/BN ProSpec® System*). Estimated glomerular filtration rate (eGFR) was calculated from serum creatinine and cystatin C levels using the CKiD-U25 equation (18). An eGFR <90 ml/min/1.73m^2^ was defined as chronic kidney disease (CKD) and then staged according to Kidney Disease: Improving Global Outcomes (KDIGO). Hyperfiltration was defined as an eGFR >140 ml/min/1.73 m^2^.

### Assessment of hypertension

Blood pressure was assessed by both office and 24-hour ambulatory measurements in patients and controls. All BP measurements were adjusted according to the normative values and expressed as their standard deviation score (SDS) values (19, 20). The office BP measurements were taken three times after a 10-min rest and then averaged. Office BP values for patients younger than 16 years were assessed according to age-, sex-, and height-specific normative values in the Fifth Report (20). For patients older than 16 years, hypertension was defined as a BP of 140/90 mmHg or above.

Twenty-four-hour ambulatory BP measurements were performed using an ABPM device (*SpaceLabs ABPM device 90217*) and an appropriate cuff. The ambulatory BP measurements of the patient group were evaluated based on the AHA criteria. Ambulatory BP measurements were classified as the following: normal BP was defined as daytime and nighttime systolic and diastolic BP <95th percentile for age and sex. Ambulatory hypertension was defined as daytime and night-time systolic and/or diastolic BP ≥95th percentile. White-coat hypertension was defined as office BP ≥95th percentile with a normal ABPM profile (21). Masked hypertension was defined as office BP <95th percentile and ambulatory systolic or diastolic BP ≥95th percentile (21). Medical history about antihypertensive treatment obtained from patient files. Patients with normal BP but under treatment with antihypertensive medication defined as controlled hypertension. Children with ambulatory, masked or controlled hypertension were defined as hypertensive.

### Kidney volume measurements

Kidney volume was measured by MRI. All MRI scans were performed at the same center and interpreted by the same radiologist. No contrast material was used and no anesthesia was administered during imaging.

**Figure 1:**
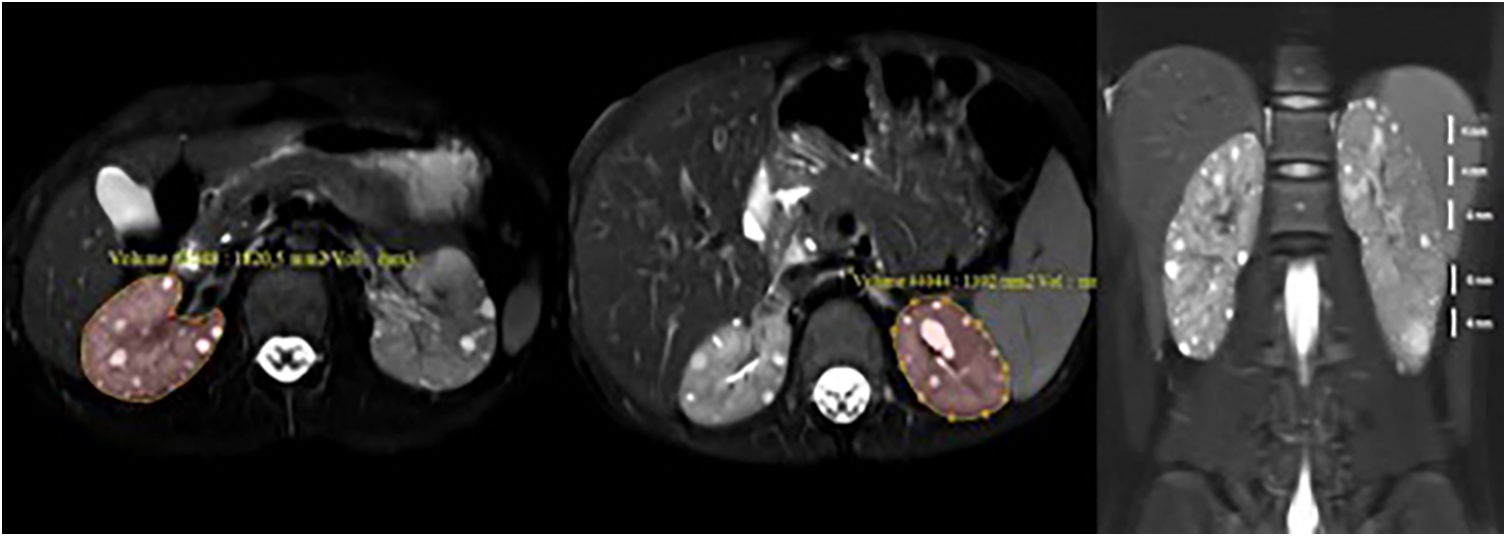
Measurement of kidney volume with MRI. Kidney volumes were determined by meticulously tracing the area on each individual slice of the axial fat-saturated T2-weighted sequence. This process involved calculating the sum of the organ areas present on each slice and then multiplying the sum by the thickness of each slice. In essence, the volume was derived by multiplying the calculated area by the height of the slices (volume = area × height). Subsequently, the calculated volumes of the right and left kidneys were summed to determine the total kidney volume (TKV).

Patients were placed in the supine position in a 3.0 Tesla superconducting MR scanner (Ingenia, Philips Medical Systems; Best, The Netherlands, 3.0 Tesla SRN:71986). The full upper abdominal MRI protocol was not applied to the subjects due to considerations of prolonged acquisition time and the primary aim of volume measurement. Consequently, axial and coronal fat-saturated T2W, and coronal mDIXON sequenced were deemed sufficient. Axial fat-saturated (FS) T2-weighted (T2W) FSE [TR/TE: 1420/80, slice thickness (st): 4 mm], coronal FS T2W FSE (TR/TE: 1270/70, st:4 mm), and coronal mDIXON (TR/TE: 1.4/3.2 st: 1.5 mm) sequences were obtained. MRI kidney volume was calculated with stereological method by measuring the area of each slice in the PACS system (22, 23). For the coronal plane T2 sequence, the area was manually drawn for each slice. The volume for each kidney was calculated using the formula “sum of calculated areas (mm2)  ×  section thickness (mm)” (section thickness =4 mm) (Figure 1). The calculated value in mm3 was divided by 1,000, and the volume was expressed as mL. These volumes of the right and left kidneys were summed to obtain the total kidney volume (TKV). This value was divided by the height (meters) to calculate the corrected kidney volume (htTKV), expressed as ml/m. Ellipsoid kidney volume was calculated using three measured orthogonal axes of the kidney in an ellipsoid equation for comparison (24).

The LIC Pediatric ADPKD model was used to categorize patients according to their htTKV into classes A, B, C, D and E. Cut-offs between severity grades of the htTKV were (ml/m) = AxB^(age^1.6) with *A* = 80, 90, 100, and 110 and *B* = 1.01, 1.012, 1.015, and 1.018 (13).

### Statistical analysis

The SPSS 20.0 program (*IBM*) was used for statistical analysis. Descriptive data for categorical variables were expressed as numbers and percentages. Continuous variables were reported as median [quartile 1 (Q1)–quartile 3 (Q3)]. The Fisher's exact test with Bonferroni adjustment was used for differences in categorical variables. Kruskal–Wallis and Mann–Whitney *U*-tests were used to compare continuous variables between multiple groups and two independent groups. Statistical significance was defined as a two-sided *p*-value of <0.05.

## Results

### Clinical characteristics

The median (Q1–Q3) age and the duration of follow-up of the 75 children with ADPKD were 6.0 (2.0–10.0) years and 3.0 (2.0–8.0) years, respectively. The male to female ratio was 1.0:1.2. As shown in Table 1, no significant differences were observed between the patient and control groups in terms of sex distribution, age, weight-SDS and height-SDS. However, the median BMI-SDS of the control group was significantly higher than that of the patient group (*p* = 0.042). A total 12 (16%) children in ADPKD group were obese. The mean eGFR of the patient group was lower than the control group but the difference did not reach statistical significance [117 (105-128) vs. 112 (100–123) ml/min/1.73 m^2^, *p* = 0.071]. A total of eight (10.7%) children with ADPKD had an eGFR below 90 ml/min/1.73 m^2^ and all of them were CKD stage 2. Hyperfiltration was found in six (8%) children with ADPKD.

**Table 1:** Characteristics of the study population.

	Control *n* = 28	ADPKD *n* = 75	*p*
Female, *n* (%)	14(%50)	34(%44)	0.83
Age, years	11.7 (9.8–12.7)	11.6 (8.7–15.7)	0.65
Weight SDS	0.68 (−0.46-1.52)	−0.99 (−0.15 to 0.85)	0.08
Height SDS	0.54 (−0.03- 1.26)	0.59 (−0.24 to 1.18)	0.92
BMI-SDS	0.22(−0.76-1.32)	−0.47 (−1.41 to 0.66)	**0** **.** **042**
eGFR (ml/dk)	123 (105–128)	112 (100–123)	0.071

SDS, standard deviation score; BMI, body mass index; eGFR, estimated glomerular filtration rate.

Values in bold indicate statistically significant results.

Data presented as median (25–75 percentile) or *n* (%).Data analyzed with the Chi-square or Mann–Whitney *U*-tests.

### Genetic findings

**Figure 2:**
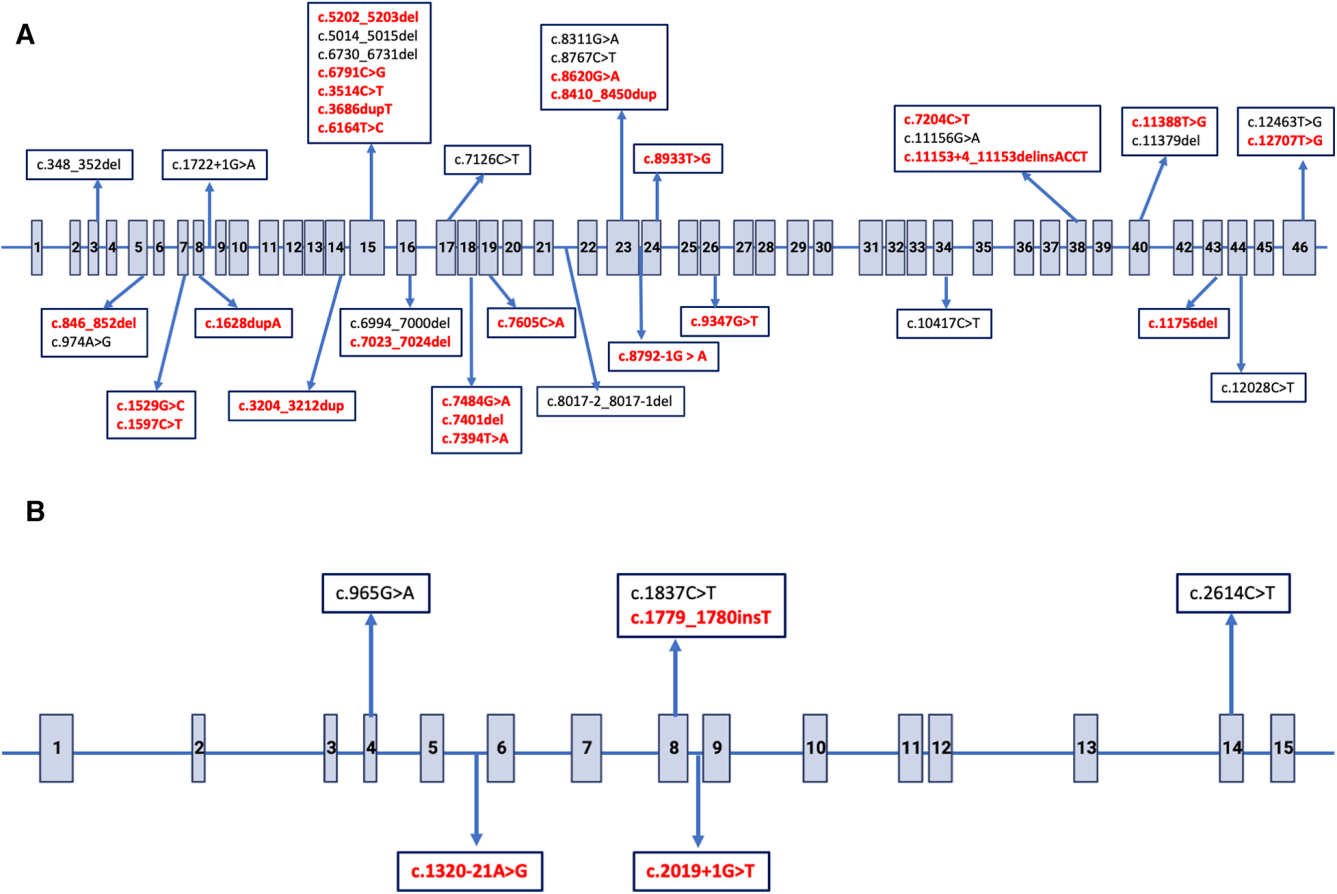
Distribution of single nucleotide variants in *PKD1* and *PKD2* gene. The arrows indicate putative positions of pathogenic variations. Novel variants are indicated with bold red. (**A**) Exon structure of the *PKD1* gene, Gen-Bank accession number NM_001009944.3. (**B**) Exon structure of the *PKD2* gene, Gen-Bank accession number NM_000297.4.

All children in patient group had a family history of ADPKD and the consanguinity rate was 6.7%. A total of 35 siblings from 17 families were included in this cohort. The percentage of patients with PKD1 and PKD2 mutations was 89.3% (*n* = 67) and 10.7% (*n* = 8), respectively. The number of children with non-missense mutations was 49 (73.1%) within PKD1 mutations and six (75.0%) within PKD2 mutations. The remaining 20 patients had missense or intronic mutations. Twenty-seven of all mutations (36%) were novel (Figures 2A,B). All mutations were shown in Supplementary Table S1.

### Blood pressure

The median systolic and diastolic BP-SDS did not differ between the patient and control groups [0.69 (0.20–1.50) vs. 0.86 (−0.03–1.62), *p* = 0.90 and 0.70 (0.24–1.22) vs. 0.98 (0.57–1.66), *p* = 0.11, respectively]. None of the BP-SDSs in ambulatory measurements (24-h MAP, daytime systolic, daytime diastolic, nighttime systolic, nighttime diastolic, systolic dipping, or diastolic dipping) differed between the patient and control groups (data not given).

The ambulatory BP measurements of the patient group were evaluated based on the AHA criteria. Of these, eight (10.7%) had ambulatory hypertension, six (8%) had masked hypertension, and 23 (31%) had white coat hypertension. In addition, 6 patients (8%) had normal BP values under ACEi/ARB for hypertension, indicating controlled hypertension. Overall, 27% of the patients (*n* = 20) were diagnosed with hypertension (ambulatory, masked, and controlled hypertension).

### Kidney volumes and risk stratification

The htTKV of the patient group were significantly higher than that of the control group [141 (113–123) vs. 117 (98–127) ml/m; *p* = 0.0003]. Patients were stratified based on the LIC for pediatric ADPKD. The number of patients in Classes A, B, C, D and E were 29 (38.7%), 21 (28%), 18 (24%), 5 (6.7%) and 2 (2.7%), respectively. Classes D and E have been combined and shown as class D + E due to the small number in these groups for statistical analysis.

Table 2 shows the comparison of the LIC classes on the basis of clinical, genetic, and BP parameters. LIC classes did not differ in terms of sex, age, height-SDS or eGFR. However, classes B, C and D + E had higher weight-SDS compared to class A (*p* < 0.05 for all), but no significant differences among themselves. Additionally, classes C and D + E had higher BMI-SDS than class A (*p* < 0.05 for both) but no significant differences were observed between each other or with class B.

**Table 2:** Clinical and genetic characteristics based on Leuven imaging classification.

LIC Severity	Class A	Class B	Class C	Class D + E	*p*
Number of patients, *n* (%)	29 (38.7)	21 (28.0)	18 (24.0)	7 (9.3)	
Female, *n* (%)	13 (46)	9 (43)	9 (50)	3 (43)	0.98
Age	12.3 (10.2–15.1)	12.4 (9.2–17.0)	9.0 (5.5–12.8)	12.4 (10.5–16.7)	0.12
Weight SDS	−0.8 (−1.1 to −0.1)	−0.1 (−0.6 to 0.7)^a^	0.9 (−0.5 to 1.4)^a^	0.4 (−0.1 to 0.9)^a^	**0** **.** **002**
Height SDS	0.3 (−0.5 to 0.8)	0.8 (0.4–1.3)	0.6 (0.2–1.9)	1.0(−0.2 to 1.8)	0.17
BMI-SDS	−1.2(−1.5 to −0.3)	−0.4(−1.4 to 0.6)	0.7(−0.7 to 1.2)^a^	0.2(−0.9 to 0.7)^a^	**0**.**011**
eGFR (ml/min/1.73m^2^)	110 (96–129)	118 (104–125)	109 (99–122)	107 (97–137)	0.90
Genetics					
PKD1, *n* (%)	25 (86)	18 (86)	17 (94)	7 (100)	0.72
NM, *n* (%)	21 (84)	10 (44)	12 (71)	6 (86)	0.19
M, *n* (%)	4 (16)	8 (56)	5 (29)	1 (14)	
PKD2, *n* (%)	4 (14)	3 (14)	1 (6)	0	0.72
NM, *n* (%)	3 (75)	2 (66)	1 (100)	0	1.0
M, *n* (%)	1 (25)	1 (33)	0	0	
Kidney volume					
Average SL, mm	91 (85–106)	103 (90–116)^a^	96 (84–106)	129 (98–144)^a^^,^^c^	**0**.**019**
Average CL, mm	94 (88–110)	102 (92–119)	98 (86–113)	133 (100–144)^a^^,^^c^	**0**.**039**
Average W, mm	40 (36–46)	50 (40–56)^a^	44 (39–57)	63 (58–78)^a^^,^^b^^,^^c^	**0**.**001**
Average D, mm	47 (43–50)	54 (44–63)^a^	49 (44–65)	69 (61–75)^a^^,^^b^^,^^c^	**0**.**001**
TKV-ellipsoid, ml	178 (146–254)	289 (166–453)^a^	233 (155–445)	604 (399–853)^a^^,^^b^^,^^c^	**0**.**002**
TKV, ml	182 (150–240)	260 (172–393)^a^	208 (149–372)	500 (352–742)^a^^,^^b^^,^^c^	**0**.**004**
htTKV, ml/m	122 (106–140)	165 (122–224)^a^	143 (121–231)^a^	338 (235–442)^a^^,^^b^^,^^c^	**0**.**0001**
Office BP					
Sys BP-SDS	0.37(−0.33 to 1.28)	0.54 (0.05–1.43)	1.60 (0.34–2.42)^a^	1.52 (1.09–1.94)^a^^,^^b^	**0**.**008**
Dia BP-SDS	0.64 (0.11–1.45)	0.93 (0.73–1.60)	1.42 (0.87–1.94)^a^	1.36 (0.80–2.03)^a^	**0**.**038**
ABPM					
24 h MAP-SDS	−0.59 (−1.24 to 0.02)	−0.39 (−0.93 to 0.46)	−0.15 (−1.05 to 0.89)	0.88 (0.41–1.22)^a^^,^^b^	**0**.**007**
Daytime Sys-SDS	−1.47 (−1.85 to −0.39)	−0.82 (−1.60 to −0.40)	−0.65 (−1.27 to 0.87)	0.88 (0.04–1.17)^a^^,^^b^	**0**.**003**
Daytime Dia-SDS	−1.02 (−1.67 to −0.60)	−0.82 (−1.27 to −0.02)	−0.45 (−1.13 to 0.27)^a^	0.34 (0.10–0.82)^a^^,^^b^	**0**.**003**
Nighttime Sys-SDS	−0.32 (−1.40 to 0.17)	−0.29 (−0.79 to 0.45)	−0.05 (−0.83 to 1.13)	0.43 (−0.06 to 1.23)^a^^,^^b^	0.123
Nighttime Dia-SDS	0.03 (−0.94 to 0.59)	0.35 (−0.46 to 1.31)	0.51 (0.12–1.07)^a^	1.25 (−0.20–2.17)^a^	**0**.**041**
Sys Dipping	10.2 (8.1–13.2)	9.4 (5.4–13.0)	11.3 (9.3–13.3)	12.9 (7.6–16.7)	0.35
Dia Dipping	17.4 (11.2–21)	16.7 (9.9–20.9)	15.1 (12.6–21.4)	13.6 (12.7–23.9)	0.98

LIC, Leuven imaging classification classes; SDS, standard deviation score; BMI, body mass index; eGFR, estimated glomerular filtration rate; PKD, policystic kidney disease; NM, nonmissense; M, missense; SL, sagittal lenght; CL, coronal lenght; W, width; D, depth; TKV, adjusted total kidney volume; htTKV, height adjusted total kidney volume.

Values in bold indicate statistically significant results.

Data presented as median (25–75 percentile) or *n* (%).

Data analyzed with the Chi-square test with Bonferonni adjustment. Kruskal–Wallis and Mann–Whitney *U*-tests were used to compare continuous variables between multiple groups and two independent groups.

^a^ Statistically different (*p* < 0.05) from the class A.

^b^ Statistically different (*p* < 0.05) from the class B.

^c^ Statistically different (*p* < 0.05) from the class C.

The evaluation of the genetic variants among children in different LIC classes showed remarkable findings (Table 2). Notably, all children in class D + E and 94% (*n* = 17) in class C had PKD1 variants. Furthermore, children with missense PKD variants were predominantly classified in class A, B or C, with only one exception in class D + E. The majority of children with PKD2 mutations (87.5%) were found in class A and B. However, these differences did not reach statistical significance.

**Figure 3:**
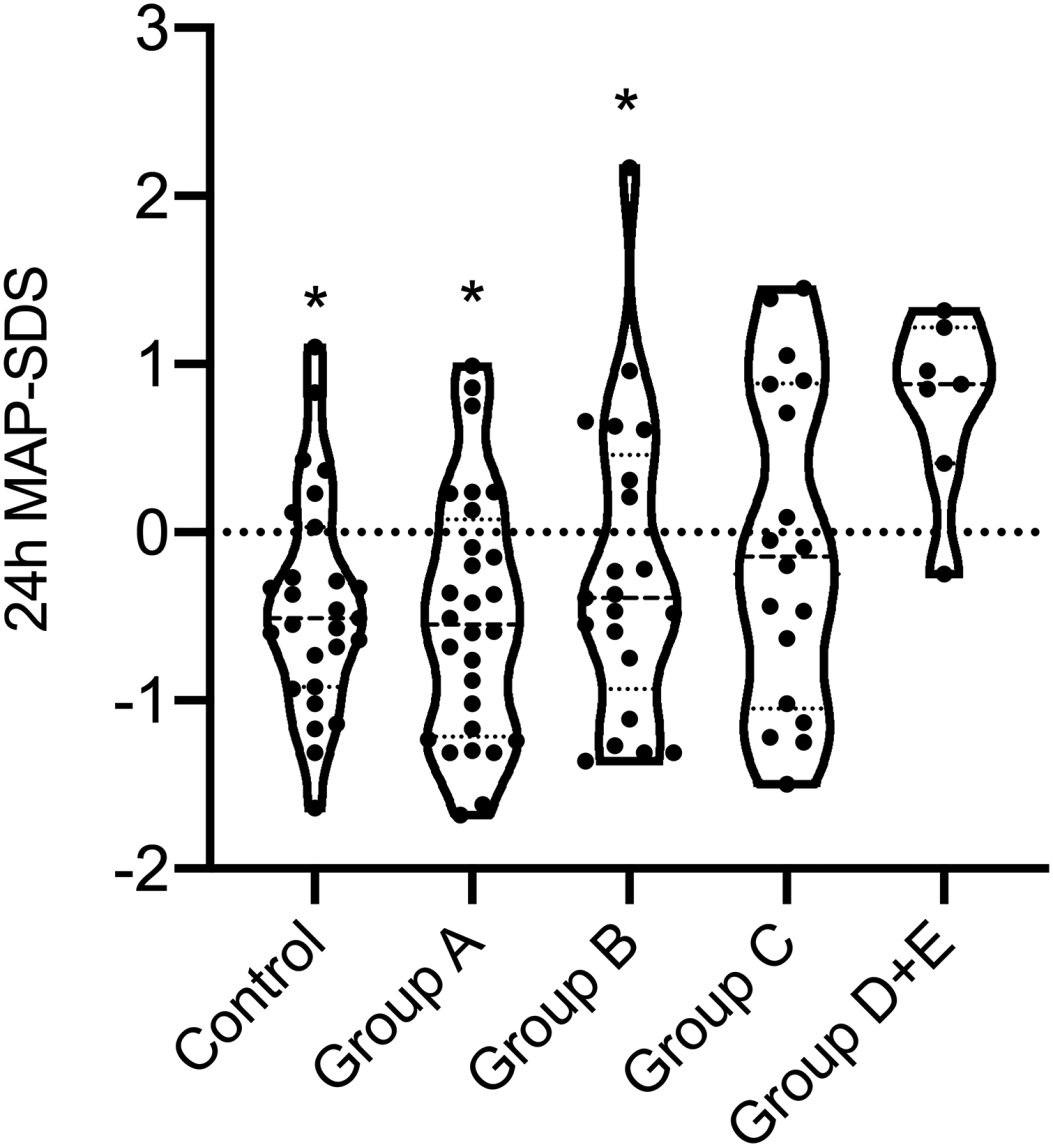
Comparison of 24 h MAP BP-SDS between LIC classes of ADPKD patients and the control group. Mann–Whitney *U*-test revealed that median 24 h MAP-SDS of class DE was significantly increased compared to control, classes A and B (*p* = 0.001, *p* = 0.001 and *p* = 0.006, respectively.) There was no difference between the other groups and control. Mann–Whitney *U*-test was used to compare continuous variables between two independent groups. * statistical significant difference compared to the class D + E.

**Figure 4:**
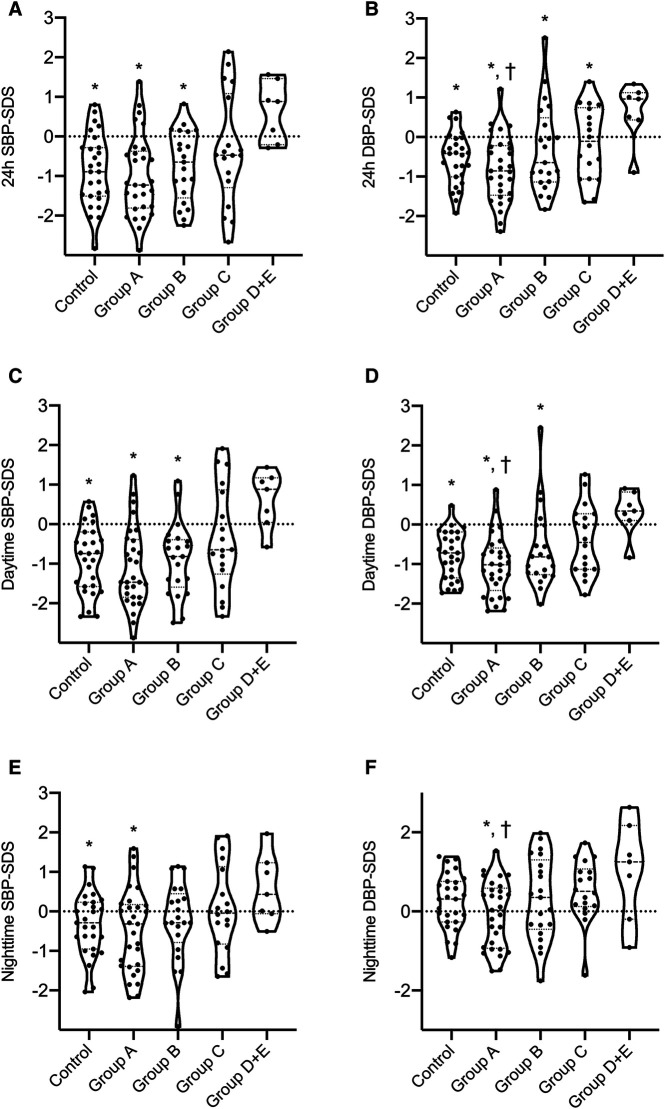
Comparison of ABPM results between LIC classes of ADPKD patients and the control group. Mann–Whitney *U*-test was used to compare continuous variables between two independent groups. * statistical significant difference compared to the class D + E. † statistical significant difference compared to the class C. (**A**) 24 h Systolic BP-SDS of class D + E was significantly increased compared to the control group, classes A and B (*p* = 0.003, *p* = 0.001 and *p* = 0.004, respectively). There was no difference between the other classes and the control group. (**B**) 24 h Diastolic BP-SDS of class D + E was significantly increased compared to the control group, classes A, B and C (*p* = 0.005, *p* = 0.027, *p* = 0.03 and *p* = 0.03, respectively). Class C had higher 24 h Diastolic BP-SDS than the class A (*p* = 0.027). There was no difference between the other classes and the control group. (**C**) Daytime Systolic BP-SDS of class D + E was significantly increased compared to the control group, classes A and B (*p* = 0.003, *p* = 0.001 and *p* = 0.002, respectively). There was no difference between the other classes and the control group. (**D**) Daytime Diastolic BP-SDS of class D + E was significantly increased compared to the control group, classes A and B (*p* = 0.003, *p* = 0.001 and *p* = 0.012, respectively). Class C had higher Daytime Diastolic BP-SDS than the class A (*p* = 0.022). There was no difference between the other classes and the control group. (**E**) Nighttime Systolic BP-SDS of class D + E was significantly increased compared to the control group and class A (*p* = 0.041 and *p* = 0.031, respectively). There was no difference between the other classes and the control group. (**F**) Nighttime Diastolic BP-SDS of class D + E was significantly increased compared to the class A (*p* = 0.023). Class C had higher Nighttime Diastolic BP-SDS than the class A (*p* = 0.026). There was no difference between the other classes and the control group.

**Figure 5:**
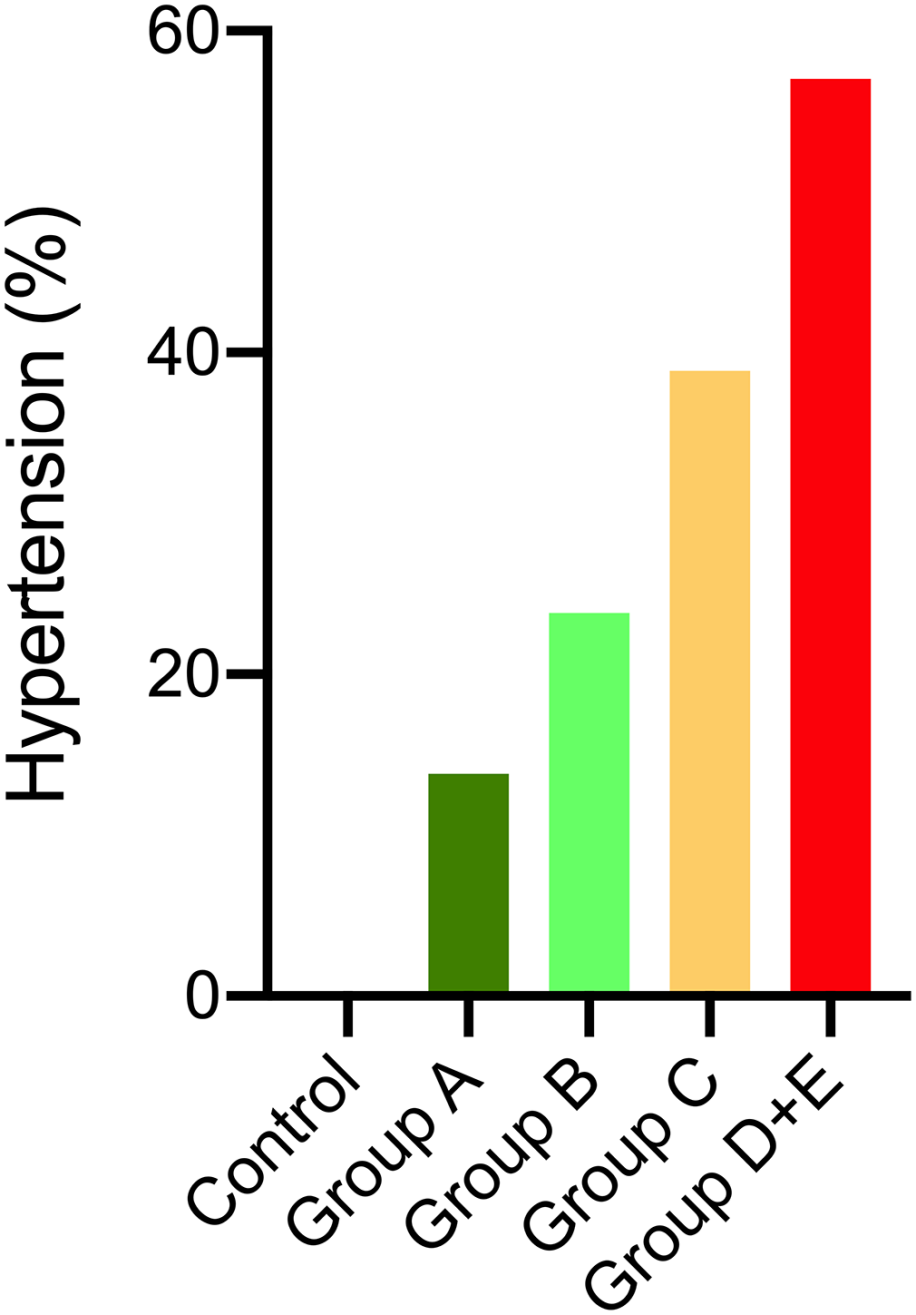
Percentage of hypertension in LIC classes of ADPKD patients and the control group. Chi-square comparison with Bonferonni showed the percentage of hypertension in classes C and D + E were higher than the control group (*p* < 0.001), but not different from classes A and B. Classes A and B did not differ than the control group.

Table 2 further shows that office systolic and diastolic BP-SDS were significantly higher in both classes C and D + E compared to class A (*p* < 0.05 for all), with class D + E exhibiting higher office systolic BP-SDS than class B (*p* = 0.023). Moreover, class D + E demonstrated significantly higher 24 h MAP-SDS, as shown in Figure 3. Class D + E also had higher daytime systolic, daytime diastolic and nighttime systolic BP-SDS than both classes A and B (*p* < 0.05, for all). Additionally, class C showed higher daytime systolic and nighttime diastolic BP-SDS than the class A (*p* < 0.05 for both). The comparison of the classes for all ABPM parameters are detailed in Table 2 and Figure 4. In classes A, B, C and D + E, the prevalence of hypertension was13.8%, 23.8%, 38.9% and 57.1%, respectively (Figure 5).

## Discussion

Our study distinguishes itself by using MRI-based measurements of kidney volume in children and the novel LIC to stratify pediatric ADPKD patients into specific risk groups. According to the stratification almost one third of these children with ADPKD fall into the three upper risk classes (C, D and E). The study also revealed a remarkable high prevalence (27%) of hypertension (HT) in children with ADPKD, with even increased rates observed in the high-risk classes (up to 56%). We also observed a higher incidence of PKD1 mutations and non-missense mutations in the high-risk classes of ADPKD.

The MIC model, a five-class severity risk model based on MRI-derived htTKV, is recognised as a validated stratification tool for adult ADPKD (7). While therapeutic trials, including the use of tolvaptan, have been initiated in adults based on the MIC, there is a lack of dedicated studies in children (<15 years) to investigate the clinical applicability of htTKV in predicting disease progression or renal function decline in adulthood. Breysem et al. (13) recently proposed a two-parameter ADPKD risk stratification model applicable to children and adolescents, incorporating htTKV and age, with validation by the Mayo and CRISP consortia using 3D ultrasound renal volumetry. Our study has shown that MRI-based renal volume measurement is also feasible in a cohort of children and young adolescents with a median age of 11.5 years. Stratification according to MRI-based htTKV showed that almost 30% of ADPKD patients classified as high-risk LIC classes, which is a lower fraction than in the Leuven cohort (54%). This discrepancy may be due to the lower number of PKD2 cases in the LIC study (4%) compared to our cohort (8%), although still lower than in the MIC study (16%) (7, 13).

The increased prevalence of hypertension in high-risk LIC classes highlights the importance of accurate blood pressure diagnosis, especially by ambulatory monitoring. While the LIC study did not show differences in office blood pressure measurements between LIC classes (13), our study showed higher blood pressure readings in both office and ambulatory measurements in children in high-risk LIC classes. Stratification of children and young people with ADPKD indicates the subpopulations at greater risk of developing hypertension.

ADPKD, which is most commonly caused by mutations in PKD1 and PKD2 genes, shows significant interindividual variability. Kidney function decline is related to the type of genetic mutation, although the rates of kidney growth do not differ between PKD1 and PKD2 (8, 25). In our cohort, all children in class D + E and most children in class C had PKD1 variants. Children with missense PKD variants were predominantly classified in low-risk classes. Phenotypic characteristics are shown to be influenced by the genotypes of the patients.

In our cohort, all children were genetically diagnosed with ADPKD. In class D + E and most children in class C, PKD1 variants were present. Children with missense PKD variants were predominantly classified in low-risk classes. While the specific gene affected and the nature of the mutation contribute to variations in the clinical presentation of autosomal dominant polycystic kidney disease (ADPKD)—with PKD1 patients experiencing more severe renal impairment and accelerated disease progression compared to those with PKD2 mutations, and PKD1 truncating mutations leading to a more severe phenotype than non-truncating mutations—it is essential to note that significant differences in the clinical characteristics of ADPKD patients cannot be entirely accounted for by the specific gene mutation (8, 25, 26).

The strength of our study lies in the utilization of a well-characterized and genotyped pediatric cohort with autosomal dominant polycystic kidney disease (ADPKD), along with the selection of MRI for kidney volume measurements. Additionally, we conducted blood pressure assessments using 24-hour ambulatory blood pressure monitoring (ABPM), providing comprehensive information on the blood pressure profiles of children in the at-risk group.

However, it is important to acknowledge the limitations of our study. While the genotype of our study population is well characterized, we recognize that the cohort size remains relatively small and limited diversity, consisting of only 75 patients and 27 controls from Türkiye. This limitation may impact the generalizability of our findings beyond the specific population. To address this concern and enhance the robustness of future research, we suggest conducting studies with larger and more diverse samples. Furthermore, our study lacks longitudinal repeated measurements to capture changes in kidney volume, kidney function, and blood pressure over time in pediatric ADPKD patients, which could offer additional insights into disease progression and the predictive value of the LIC model. Lastly, a notable limitation is the non-routine use of MRI-based measurements, especially in small children, necessitating the incorporation of correction factors for 2D ultrasound in daily practice.

In conclusion, our research validated the innovative LIC ADPKD stratification model within a finely characterized and genotyped pediatric ADPKD cohort using MRI-based renal volume measurements. Notably, children classified in higher LIC risk categories had an increased incidence of hypertension and a higher prevalence of PKD1 mutations. The use of MRI in this pediatric population was found to be feasible, promising validity and continuity in full age spectrum. The essential role of ambulatory blood pressure monitoring in identifying hypertension in high-risk groups was highlighted. By emphasizing the importance of classifications, our findings highlight their crucial role in identifying and selecting high-risk patients for potential treatments and inclusion in clinical trials.

## Data Availability

The original contributions presented in the study are included in the article/Supplementary Material, further inquiries can be directed to the corresponding authors.
